# Teaching Health Education Through the Development of Student Centered Video Assignment

**DOI:** 10.3389/fpubh.2019.00312

**Published:** 2019-11-01

**Authors:** Heather Wallace, Julia VanderMolen

**Affiliations:** Public Health Department, Grand Valley State University, Grand Rapids, MI, United States

**Keywords:** health education, pedagogy, undergraduate education, multimedia, public health competencies

## Abstract

The purpose of this study was to explore the ways in which a student centered video assignment enhanced student learning about developing and teaching a health education plan designed to address a complex public health problem. The objectives of the assignment included (1) to explore a complex public health problem, (2) practice developing a corresponding health education plan, and (3) develop and fully execute a multimedia video to deliver high impact health education and or promotion to a diverse audience. The assignment referred to as the student centered video assignment was developed and piloted at Grand Valley State University and included students participating in a 200 level undergraduate introductory public health course. Students working in groups of 3, worked through the project in multiple phases with corresponding elements: problem identification, problem solving, plan development and the creation of the video, which included four primary elements: Dig Deeper, Think, Discuss, and Watch. Upon completion of the video, students were asked to voluntarily complete a 10-question survey about the benefits of learning through a video creation assignment. A total of 15 students completed the survey with the majority either strongly agreeing or agreeing that the video assignment enhanced learning. Survey results suggest that the assignment is beneficial and favored over other assignment types. Additionally, students reported that the project was beneficial in learning the concepts and competencies associated with public health education. Finally, students indicated that the creation of videos as a means to explore and apply course concepts was favored as an assignment format in future coursework. Instructor assessment of learning outcomes occurred through ongoing grading of and feedback on the project elements. The findings reveal that students enjoyed the opportunity to engage in real world problem solving, gained insight, perspective and scope through group discussion and collaboration, and felt that they learned new skills in created the video.

## Background and Rationale: Multimedia Technology and Higher Education Pedagogy in Public Health and Health Science

Changes in technology are rapidly changing our culture (1). New teaching and learning formats that rely on digital and web-based technologies have greatly influenced how instructors teach as well as how students learn (2). Resnick (3) of Massachusetts Institute of Technology's Media Laboratory (MIT Media Lab) describes our society as rapidly transforming into a creative society, one in which the skilled and expert workers of the past century are replaced by creative workers adept at problem-solving. Such a trend suggests the importance of creativity in student learning and emphasizes that problem solving is not only a critical skill, but also one that can be facilitated through technological tools. Technology can enhance effective learning in many ways (4–6), such as providing greater depth of functionality, feedback, interactivity and simulation (2). A bank of empirical research demonstrates that blogging, video blogging (vlogging) and podcasting, when used as a part of classroom instruction, can enhance student performance, foster reflection, creativity, and knowledge construction (7, 8). Likewise, students can be instructed to create their own multimedia work as part of a learning activity or assignment. The work of Fredenberg (9) and Armstrong et al. (10) provide examples of how students can create podcasts, rather than listening to them as an instructional tool. Fredenberg (9) reported that students are more engaged and felt more confident in their skills and abilities after mastering a podcast assignment. Armstrong et al. (10) required student-created podcasts (students could elect audio vs. video) as a means to learn objective driven business communication. Having students produce their own podcasts provided a novel way for students to disseminate information while also developing communication and literacy skills through teamwork, organization, time management, technical literacy, and overall planning (10).

The core competencies for public health professionals and other health care providers rest heavily on communication skills, literacy, creativity, problem solving, and the appropriate use of technology. Having students explore, create, and disseminate through video and other multi- media formats, such as podcasts and vlogging, may offer a novel and effective tool for enhancing these skills. Despite the growing body of research and popularity of multimedia in higher education, health education, and society, little scholarship has addressed the use of student created video and the pedagogical benefits specifically for health science students. This paper seeks to offer a starting point for exploring how student created videos can enhance health science and public health education.

## Pedagogical Framework and Principles: Student Created Videos in Problem Based Learning

Students engaged in health profession education encounter numerous “wicked problems” that require teaching and learning strategies that allow for flexibility, trial and error, and engagement beyond text book and lecture based learning. The student centered video assignment was developed, in part, as a strategy to facilitate this type of learning in undergraduate courses with a public health focus, such as Introduction to Public Health. The course was taught by a faculty member from the Grand Valley State University, College of Health Professions, Master's of Public Health (MPH) Program (MPH). The student centered video assignment was developed jointly by the authors, each having over 10 years of teaching experience in the health professions. The course is offered each semester in multiple sections and has been taught multiple times by each of the authors. The class utilized a traditional in seat format while using inquiry based learning as the pedagogical approach. This approach challenges students to learn through directed questions, problems, and challenges that students work to address. Additionally, the courses incorporated Problem Based Learning (PBL) which is known as a teaching and learning approach that facilitates cultivation of knowledge, critical thinking, assessment and evaluation in solving complex, real world problems. Students engaged in PBL set to exploring and solving problems in small groups with the objective of promoting “constructive, self-directed, collaborative and contextual activity” (11). PBL research in nursing and clinical education showed improvement in critical thinking and critical reasoning (12), while research on the *process* of PBL reflects gains in collective and collaborative knowledge building (13).

### Principles and Competencies in Health Education and Promotion

The Council on Education for Public Health (CEPH) is the accrediting body for all schools and programs of public health in the United States. They have identified 22 primary core competencies for public health students and professionals. Of the 22 competencies identified by CEPH, 6 were used in the organization of the two courses in which the student centered video assignment was implemented (Table 1). The competencies selected align with the course objectives and public health program concepts. Execution of the student centered video assignment provides ample opportunity for students to practice and build competency in these areas. For example, competency 16, 21, and 22 is addressed through group collaboration and working with a complex public health issue. Competency 18, 19, and 20 is achieved through the creative development of the video, in which a public health message and health promotion tool are developed for a target population (Table 1).

**Table 1:** CEPH competencies used in course and assignment development.

**CEPH course competency**	**Description**
16	Apply principles of leadership, governance and management, which include creating a vision, empowering others, fostering collaboration, and guiding decision making
18	Select communication strategies for different audiences and sectors
19	Communicate audience-appropriate public health content, both in writing and through oral presentation
20	Describe the importance of cultural competence in communicating public health content
21	Perform effectively on interprofessional teams
22	Apply systems thinking tools to a public health issue

Achieving competency requires meaningful student learning and mastery of skills related to analysis, assessment, planning, communication, management, and systems thinking (14). Video creation using a problem based learning approach allows for students to practice these skills through engaging with a real world problem in small groups and to explore and practice mastering a new technology.

## Methods

### Learning Environment

Grand Valley State University is a midsize, teaching intensive University located in the Midwest. The College of Health Professions houses 17 undergraduate and graduate health profession programs. The Department of Public Health offers a Master's Degree in Public Health (MPH) and includes several introductory undergraduate courses. The student centered video assignment was created by the authors who are faculty from the MPH program who teach undergraduate and graduate public health courses. The goal was to create a novel course learning activity that facilitated innovative and collaborative development of course work that is student centered, linked to real world public health issues, problem focused and outside of traditional course projects, such as research papers and or presentations. This assignment was developed and piloted in a 200-level undergraduate introductory public health course Students are generally traditional undergraduate college students at the sophomore or junior level interested in exploring a health related degree and or career. The undergraduate public health courses provide a valuable opportunity for undergraduate students to gain awareness and knowledge of the public health profession and the MPH degree. The students in the course that utilized the student centered video assignment are representative of the larger undergraduate student body. Further, students enrolled in the course as well as the students that completed the course evaluation and assignment evaluation reflect a general health professions student body that is disproportionately female.

### Video Creation Assignment

The video creation assignment was developed and piloted in the 15 weeks fall semester of 2017. The overall purpose of the assignment was to provide students with a robust, collaborative, innovative, learning opportunity in which a complex public health problem are identified, explored, and responded to through the creation of a culturally appropriate health education video. The assignment was completed in four phases over a 12 weeks period, with each phase having an associated learning element. The phases and elements for the assignment are informed through the PBL pedagogical approach of the course as well as the CEPH competencies listed in Table 1. The following sections describe the objectives and course work associated with each phase and for phase 4, each element of the student centered video assignment.

#### Phase 1

The objective of phase 1 is to introduce and facilitate student exploration of real world public health problems. In order to achieve this objective students are randomly assigned to groups of 3–4 depending on the number of students in the course. Over a 3–4 weeks period of time, students are given access to course materials through the University learning platform which include scholarly research articles, journalistic documentaries, and web resources that reflect ongoing and emergent public health problems that are salient to health promotion and or health education. For example, students review topics like vaccine preventable infections and outbreaks, drug use during pregnancy, harm reduction measures among homeless and drug addicted youth, vaping, and sleep hygiene among college students. Students explored the problems together in class and through consensus, elected a problem of interest that is explored and analyzed in Phase 2.

A second learning activity in Phase 1 is the introduction of and experimentation with video creation technology. Students viewed professionally developed TED-Ed video talks, which served as an exemplar and model for the structure of the student created video. Students were asked to watch health related Ted-Ed talks and to reflect upon the message, format, effectiveness, and efficiency of the message.

#### Phase 2

Class time during weeks 4–6 is used to discuss and describe how to explore, evaluate, and assess root causes and systems level factors related to the selected public health problem. The objective of Phase 2 is to use principles of descriptive Epidemiology, such as time, place and person, to explore the natural history, context, and scope of the selected public health problem. In this phase, the first element, “Dig Deeper,” is introduced.

The public health problem identified in Phase 1 becomes the focus of the Dig Deeper Element introduced in Phase 2. Students are asked to dig deeper into their problem by searching for and evaluating relevant and credible literature and web resources. Students were provided with a website evaluation tool, the CRAAP, to assist in reviewing web information (15). Each group member composed a single paragraph between 250 and 500 words that included a minimum of three credible references and summarized their search and evaluation. Group members then worked collaboratively to combine the paragraphs into a concise health education or health promotion message relevant to their public health problem. As an introductory undergraduate course, gaining scope and depth of resources accessed and utilized was not the intention. Rather, the dig deeper element was considered a skill building opportunity for group collaboration and health information seeking, assessing and summarizing. Basic instructions for the Dig Deeper Element are provided in Appendix A.

#### Phase 3

The development of a video health education or health promotion plan is the objective of Phase 3. The plan becomes the framework for the actual video that the students produce in Phase 4. The basic format of the plan is aligned with the TED-Ed lesson creation guide and consists of a short informative message derived from the Dig Deeper element, thinking questions related to the message, a learn more section and a discussion section (16). The first step of Phase 3 is to complete the “Think Element,” in which students were required to create a series of multiple choice and true or false questions, which addressed the content from the Dig Deeper Element. The questions check for understanding of the content presented in the Dig Deeper element. Students were encouraged to develop thought-provoking questions at a Bloom's level of Analysis or higher. Basic instructions for the Think Element are provided in Appendix B.

The second step of Phase 3 is the Discuss element. The Discuss element required each student within the group to compose a thoughtful discussion question pertaining to the information provided in the Dig Deeper element and complimentary to the Think element. The purpose of the Discuss element is to provide an opportunity for viewers of the video to discuss and further explore the topic of the video. Students were encouraged to review *Guidelines for Developing Juicy Discussion Questions* (17). Groups were also encouraged to create a Google Doc so that each student could peer review and edit the discussion questions. Students were limited to 750-character limit. Basic instructions for the Think Element are provided in Appendix C.

#### Phase 4

In phase 4, student groups developed and executed TED-Ed like educational videos using the Watch element. Student groups were required to create an original video incorporating the Dig Deeper, Think, and Discuss elements they had completed in phases 1–3. Groups applied their topic research, questions, and discussion content to a storyboard template provided by the instructor. The instructor provided students with access to PowToons for Education as the medium or technology used for creating the actual video. PowToons offers a free educational subscription to web-based animation software designed to create animated videos (18). The videos were ~5 min long and were presented in class on the last day Basic instructions for Phase 4 or the Watch Element are provided in Appendix D.

### Setting and Participants

The setting for the student centered video assignment was an in seat undergraduate introduction to public health course with 48 students. Two of the 48 students were male and 46 were female. All students in the course completed the assignment as part of the course work.

Student feedback and learning related to the student centered video assignment was gathered through an evaluation. As the assignment was part of the course, informed consent for participating in the assignment was not needed nor obtained. Informed consent was obtained for the evaluation of the assignment that took place following the viewing of the videos in the last class meeting. The Grand Valley State University Institutional Review Board (IRB) approved the evaluation and deemed the study exempt. The evaluation of student perception of the student-centered video assignment included 10 Likert scaled questions (rating questions 1–7, 1 strongly disagree-−7 strongly agree.) Forty-eight students were emailed the survey following the last day of class. Fifteen students completed the survey for a 31% return rate.

## Results

Of the 15 students who completed the survey the majority either strongly agreed or agreed that the video assignment enhanced learning. Survey results suggest that the assignment is beneficial and favored over other assignment types, such as traditional course research papers and or oral paper or poster presentations. Likewise, students would prefer the video assignment over other types of assignments in future classes. Additionally, students reported that the project helped them to apply and develop understanding of the concepts and CEPH competencies associated with introductory public health education. A total of 86% of the students indicated that the student-created video lesson enhanced their learning of public health content. Additionally, 73% of the students thought that creating videos to address a public health issue was a useful experience. Seventy-three percent also indicated that the student-centered video assignment was an important exercise in helping them better understand critical public health issues. Table 2 and reflect the survey questions and student response regarding the student centered video assignment.

**Table 2:** Student responses to the TED-Ed student-centered video assignment.

**Question**	**Strongly agree**	**Agree**	**Somewhat agree**	**Neither agree nor disagree**	**Somewhat disagree**	**Disagree**	**Strongly disagree**
Creating videos in class is an important exercise	13.33% (*n* = 2)	33.33% (*n* = 5)	26.67% (*n* = 4)	13.33% (*n* = 2)	6.67% (*n* = 1)	6.67% (*n* = 1)	0.00% (*n* = 0)
Creating videos in class is an enjoyable experience	6.67% (*n* = 1)	33.33% (*n* = 5)	33.33% (*n* = 5)	6.67% (*n* = 1)	13.33% (*n* = 2)	6.67% (*n* = 1)	0.00% (*n* = 0)
Creating videos is a useful experience	13.33% (*n* = 2)	53.33% (*n* = 8)	6.67% (*n* = 1)	13.33% (*n* = 2)	6.67% (*n* = 1)	6.67% (*n* = 1)	0.00% (*n* = 0)
Creating videos is an interesting experience	20.00% (*n* = 3)	46.67% (*n* = 7)	20.00% (*n* = 3)	6.67% (*n* = 1)	6.67% (*n* = 1)	0.00% (*n* = 0)	0.00% (*n* = 0)
The video project was the most useful project in class	6.67% (*n* = 1)	6.67% (*n* = 1)	33.33% (*n* = 5)	26.67% (*n* = 4)	26.67% (*n* = 4)	0.00% (*n* = 0)	0.00% (*n* = 0)
I would rather do a different project than the video project	0.00% (*n* = 0)	13.33% (*n* = 2)	6.67% (*n* = 1)	6.67% (*n* = 1)	33.33% (*n* = 5)	40.00% (*n* = 6)	0.00% (*n* = 0)
Participating in the video project will help my career	6.67% (*n* = 1)	33.33% (*n* = 5)	13.33% (*n* = 2)	33.33% (*n* = 5)	6.67% (*n* = 1)	6.67% (*n* = 1)	0.00% (*n* = 0)
Creating videos enhances learning content	26.67% (*n* = 4)	46.67% (*n* = 7)	13.33% (*n* = 2)	6.67% (*n* = 1)	0.00% (*n* = 0)	6.67% (*n* = 1)	0.00% (*n* = 0)
Creating videos will enhance learning in future classes	26.67% (*n* = 4)	40.00% (*n* = 6)	20.00% (*n* = 3)	6.67% (*n* = 1)	0.00% (*n* = 0)	6.67% (*n* = 1)	0.00% (*n* = 0)
I would recommend this project for future classes	33.33% (*n* = 5)	46.67% (*n* = 7)	6.67% (*n* = 1)	6.67% (*n* = 1)	6.67% (*n* = 1)	0.00% (*n* = 0)	0.00% (*n* = 0)

It is not clear whether the video format or the PBL learning approach associated with the assignment influenced student preferences and responses. However, this initial evaluation along with instructor evaluation of the assignment elements completed by the groups, suggests that students enjoyed the opportunity to engage in real world problem solving, gained insight, perspective and scope through group discussion and collaboration, and felt that they learned new skills in created the video.

## Discussion

Overall, this project received strong and positive evaluations from students that serve as a starting point for further development, use and evaluation of similar video creation assignments. A primary strength of this assignment and preliminary evaluation is that it offers credibility and feasibility of student-created videos as a learning tool in higher education. In addition to offering a fun, innovative and novel skill set in the way of video creation, the assignment may offer valuable means for students to develop skills in problem solving and collaboration. Such skills are necessary in the modern workplace where sophistication and complexity of problems is evolving and changing. Furthermore, the video assignment was perceived to be useful in addressing key competencies related to public health and health education which are also becoming more ambiguous and complex. Limitations to the study include the moderately sized sample of students as well as the limited number of students who completed the survey. Furthermore, the generalizability of these findings is limited by the fact that this research was conducted within a single university and a single course.

This assignment serves as an example of an innovative and complex assignment with implications and consequences related to learning but also to achieving high impact learning at the undergraduate level. In other words, undergraduates undertaking this assignment are forced to confront uncomfortable and often controversial public health issues that have no clear good or bad, right or wrong answers. Success is not dependent upon memorization of course concepts but on a students ability and willingness to identify and confront their own learning styles, expectations, and collegiality in working with the instructor, class mates, group members and tangentially, the population of people their video is meant to address.

### Recommendations for Educators

The experience of the assignment from the perspective of the instructor offers guidance and caution for other educators willing or curious to take on such an assignment. First, explicit instructions along with a rationale should be given to students at the outset of the semester or work period. The basic components of a student groups, a real life problem related to the course topic and or discipline and a strategy for the development of video contents and video creation are all that are needed to develop a similar assignment. However, it should be noted that students in the course conveyed concern over the timeline, uncertainty in understanding assignment expectations, and in some cases, wanted to jump ahead in creating a video without utilizing the phases and elements. Providing a detailed map of the project ahead of time may ease student concern and better facilitate dedicated in class work time. In this regard, instructors should be willing and able to dedicate a significant portion of in seat class time (45–60 min per element) to group work in completing the elements of assignment. Other aspects of the assignment, such as accessing and viewing TED-Ed lessons and public health resources can be facilitated through an online course delivery platform, such as Moodle or Blackboard. A second recommendation for educators is to seek ways to extend the work of the video creation outside of the classroom. The ability to showcase and “test out” the video with a real target population was requested by the students. The ability to self-select a problem and to work on it in a deliberate way appears to raise interest and investment in the project among students. Finally, as an assignment fully entrenched in collaborative group work, care and consideration should be given to address students who are either uncomfortable or unwilling to engage fully and fairly in group work. Likewise, the instructor should acknowledge, address, and provide a model for handling disagreement within groups. A policy, for example, included in the course syllabus could provide a pathway for students to address concerns among themselves prior to contacting the instructor. The use of a self and peer evaluation that is included in the final course grade may also provide incentive or meaningful consequence for students who do not participate fully or underperform in the group work.

### Instructor Reflection on Evaluation of the Assignment

The instructor reviewed and provided feedback to groups upon completion and submission of each element within each phase of the project. The video assignment work was intentionally designed to be low stakes, meaning that groups received a complete or incomplete for each element with the expectation that feedback given would be considered in the ongoing work, resulting in a strong and appropriate video. Only the final video was scored and included the self and peer evaluation. In this way, each group member receives his or her own unique score rather than a base score for the group. The evaluation matrix used for the final video displayed in class included assessment based on the 4 elements and the CEPH competencies. This included the representativeness of the information provided in the Dig Deeper element; the complexity and appropriateness of the Think element and Discussion questions; and the overall aesthetic value and quality of the video, it's length, and it's overall message. Graduate level courses could significantly increase the complexity and scope of the evaluation matrix to include elements, such as health and cultural literacy, sophistication, timeliness and appropriateness of the literature and resources used to develop the health education plan, and pre- and post-test elements to gauge viewer learning. Finally, anectodal evidence from student work, comments and discussion offer further support for this type of assignment. Students provided thoughtful, well-written, well-supported work that reflected the healthy struggle of reaching group understanding and agreement and to conceptualize difficult and complex problems.

Further studies should examine more closely the specific characteristics of the assignment that may cultivate benefit in learning and how student centered, problem based, collaborative course work around a short, audio visual rather than written assignment can be useful in attaining high level learning outcomes.

## Conclusion

The use of a student centered video assignment may be a novel and innovative strategy to approach the development of problem solving and group work in the classroom. The assignment described here was received positively by students and offers insight into the myriad complexities of teaching about difficult, evolving, and sophisticated issues both in and outside of the health professions. The small but positive results of the student survey as well as the assessment of student learning witnessed by the instructor should encourage other educators to develop similar assignments and to undertake additional studies to evaluate the effectiveness, merits, and strengths of creating video in public health coursework.

## Ethics Statement

This study was carried out in accordance with the recommendations of Grand Valley State University Institutional Review Board with written informed consent from all subjects. All subjects gave written informed consent in accordance with the Declaration of Helsinki. The Grand Valley State University Institutional Review Board approved the protocol.

## Author Contributions

JV contributed to the conception and design of this study. JV managed the data collection of this study. JV organized the data. HW and JV wrote the first draft of the manuscript. JV and HW wrote the sections of the manuscript. HW wrote the second draft of the manuscript and completed all revisions.

### Conflict of Interest

The authors declare that the research was conducted in the absence of any commercial or financial relationships that could be construed as a potential conflict of interest.
