# Supplementary material for: Teaching Health Education Through the Development of Student Centered Video Assignment
Source: Front Public Health. 2019 Nov 1;7:312. doi: 10.3389/fpubh.2019.00312 (PMC6839524; doi:10.3389/fpubh.2019.00312)
Supplement: Supplementary file 1 [file Data_Sheet_1.docx]

Appendix A

TED-Ed Dig Deeper Element Instructions

**Instructions:**

1. One member of the group will create the Google Doc.
2. Name the Google Doc with the last name of the group members and TED-Ed Dig Deeper.
3. Be sure to share the doc with the other group members.
4. Be sure to share with your instructor.

**Criteria:**

1. Each group member will compose a paragraph. The paragraph should be a minimum of 250 words but no more than 500. Each member will locate and review three outside resources for the paragraph.
2. Create hyperlinks to the resources, which are credible.
3. Be sure to provide the APA citation reference page at the end of the Google Doc. The reference list does not need to be in the Dig Deeper portion of the TED-Ed.

**Submission**

1. Access Blackboard and go to the Learning Hub>Assignment and Exams>TED-Ed Dig Deeper Submission Link
2. In the comment area be sure to include the following:
   1. The last name of each group member
   2. The Google Doc share link
   3. Be sure to make the link active
3. Only one group member needs to submit the Share link from the Google Doc.

Appendix B

TED-Ed Think Element Instructions

**Instructions:**

1. One member of the group will create the Google Doc.
2. Name the Google Doc with the last name of the group members and TED-Ed Think.
3. Be sure to share the doc with the other group members.
4. Be sure to share with your instructor.

**Criteria**

Each group member will create two multiple choice or true false questions. Please be sure to use proper grammar and check for spelling.

**Submission**

1. Access Blackboard and go to the Learning Hub>Assignment and Exams>TED-Ed Think Submission Link
2. In the comment area be sure to include the following:
   1. The last name of each group member
   2. The Google Doc share link
   3. Be sure to make the link active
3. Only one group member needs to submit the Share link from the Google Doc.

Appendix C

Ted-ED Discuss Element Instructions

**Instructions:**

1. One member of the group will create the Google Doc.
2. Name the Google Doc with the last name of the group members and TED-Ed Discuss.
3. Be sure to share the doc with the other group members.
4. Be sure to share with your instructor.

**Criteria:**

1. Each group member will compose a discussion question.
2. Students are encouraged to review the following pdf from Edutopia.

<https://www.edutopia.org/pdfs/stw/edutopia-stw-assessment-high-sch-humanities-discussion-questions-guide.pdf>

1. Grammar and spelling will be reviewed.

**Submission**

1. Access Blackboard and go to the Learning Hub>Assignment and Exams>TED-Ed Discuss Submission Link
2. In the comment area be sure to include the following:
   1. The last name of each group member
   2. The Google Doc share link
   3. Be sure to make the link active
3. Only one group member needs to submit the Share link from the Google Doc.

Appendix D

PowToons and TED-Ed Watch Instructions

**Instructions:**

1. Groups will create an original video about the selected public health topic.
2. One member of the group will create the PowToons account
3. Using PowToons, groups will create a video no more than five minutes in length. Minimum of at least two minutes in length pertaining to a public health issue.
4. One person in the group will upload the video to YouTube.
